# Potential Intersections between lncRNA, Vascular Cognitive Impairment, and Immunization Strategies: Insights and Future Directions

**DOI:** 10.3390/vaccines12030251

**Published:** 2024-02-28

**Authors:** Yishu Fan, Bo Xiao, Mengqi Zhang

**Affiliations:** 1 Department of Neurology, Xiangya Hospital, Central South University, Changsha 410008, China; fanyishu@csu.edu.cn (Y.F.); xiaobo_xy@126.com (B.X.); 2 National Clinical Research Center for Geriatric Disorders, Xiangya Hospital, Central South University, Changsha 410008, China

**Keywords:** vascular cognitive impairment (VCI), long non-coding RNA (lncRNA), next-generation sequencing (NGS), immunology, vaccine

## Abstract

Vascular cognitive impairment (VCI) encompasses a wide range of cognitive disorders stemming from cerebrovascular issues, such as strokes or small vessel disease. These conditions often pose challenges to traditional diagnostic approaches due to their multifactorial nature and varied clinical presentations. Recently, next-generation sequencing (NGS) technologies have provided detailed analyses of long non-coding RNAs (lncRNAs) in the molecular pathobiology of VCI. These new findings help with molecular-based diagnostics and treatments of VCI. Within this realm, the concept of immune modulation, especially through specific vaccinations, emerges as a promising therapeutic strategy in VCI mitigation. In this review, we comprehensively elucidate the function of lncRNAs in VCI, emphasizing the advanced understanding of VCI’s molecular underpinnings made possible through NGS technologies. Significant focus is placed on the immune system’s role in VCI, particularly the neuroinflammatory processes which are consequential to cerebrovascular abnormalities. We believe that lncRNAs participate in regulating these immunological pathways, potentially guiding the development of vaccines targeting VCI. In this context, we propose a novel perspective: using knowledge about lncRNA profiles and functions to guide vaccine development, we can potentially exploit the body’s immune response to mitigate or prevent VCI. This approach has the potential to revolutionize VCI management by introducing targeted immunization strategies informed by molecular signatures, a concept that remains largely unexplored in current research endeavors. In addition, we summarize current progress and propose future directions, advocating for robust, interdisciplinary studies to validate the potential intersections between lncRNA landscapes, VCI pathology, and immunology. This review aims to spur innovative research and promote the development of lncRNA-informed vaccine strategies as proactive interventions against the cognitive consequences of VCI.

## 1. Introduction

Vascular cognitive impairment (VCI) includes various disorders in which vascular factors lead to diminished cognitive functions. This category encompasses a range of conditions, including vascular diseases like multi-infarct dementia, along with mild cognitive impairment falling short of dementia diagnosis. It also includes cognitive decline involving a combination of vascular and degenerative factors [1]. With the aging population on the rise in China, there has been a noticeable increase in dementia cases. Research indicates that around 20.8% of individuals aged 65 and older experience mild cognitive impairment (MCI) [2], with a substantial 42.0% of these cases being linked to cerebrovascular diseases and vascular risk factors. Furthermore, among all dementia cases, vascular dementia (VaD) accounts for about 20%, ranking as the second most frequent form following Alzheimer’s disease (AD) [3]. The increasing prevalence of dementia among China’s aging population poses significant challenges. These challenges affect not only patients and their families but also place a heavy burden on societal resources and healthcare infrastructure. Urgent attention and innovative solutions are required to address this issue effectively. Studies have shown that the risk factors for VCI include alterable and unchangeable factors, such as age, ethnicity, and genetic predispositions [4]. Currently, it is known that featured pathological changes in VCI patients include white matter lesions, cerebral infarctions, and hemorrhages that cause tissue damage and loss. However, further clarification is required regarding these details, primarily due to the overlapping characteristics shared by VCI, stroke, and Alzheimer’s disease pathology [5]. Meanwhile, the limited understanding of the molecular mechanisms driving the onset and progression of VCI continues to limit the development of VCI treatment.

In recent years, societal advancements and technological progress have spurred the development of a myriad of innovative technologies. Among these significant technological advancements, next-generation sequencing (NGS) stands out for its transformative impact, especially in the realms of biology and medicine [6]. NGS refers to a group of advanced sequencing technologies that involves the high-throughput parallel sequencing of millions of DNA or RNA fragments simultaneously [7]. This method is significantly faster and more cost-effective than traditional Sanger sequencing [8]. NGS allows for the cost-effective comprehensive analysis of genetic sequences at an unprecedented scale, facilitating a deeper understanding of the genetic variations of complex genetic diseases and molecular mechanisms underlying various biological processes. NGS is extensively utilized across various areas within biomedical science, encompassing areas such as genetics [9], infectious diseases [10], oncology [11], personalized medicine [12], drug development [13], and preventive medicine [14]. By enabling the detailed study of genomes and gene expression, NGS is revolutionizing our approach to diagnosing and treating diseases, understanding genetic disorders, and exploring the complexities of the human genome. At present, advancements in high-throughput sequencing technology have enhanced the research on non-coding RNA (ncRNA), revealing that long non-coding RNA (lncRNA) plays a crucial role in the onset and progression of VCI [15].

LncRNAs are a class of non-coding RNAs typically longer than 200 nucleotides in length. These molecules are highly abundant and exhibit tissue-specific expression patterns, suggesting their importance in cellular homeostasis and development. Despite their lack of protein-coding capacity, lncRNAs play crucial roles in regulating various cellular processes and gene expression in epigenetic, transcriptional, and post-transcriptional processes [16]. LncRNAs can exert their regulation functions through diverse mechanisms, including acting as scaffolds for protein complexes, guiding chromatin-modifying complexes to specific genomic loci, modulating mRNA stability, and functioning as competitive endogenous RNAs (ceRNAs) by sequestering microRNAs [17]. LncRNAs are also capable of directly influencing epigenetic, transcriptional, post-transcriptional, and chromatin remodeling stages, particularly through RNA splicing and similar mechanisms [18]. Additionally, functioning as competitive endogenous RNAs (ceRNAs), lncRNAs have the ability to combine with microRNAs (miRNAs), thereby influencing the regulation of target proteins [19]. In addition, lncRNAs play crucial roles in several biological activities such as apoptosis, angiogenesis, cell differentiation, immune response, autophagy, and cell proliferation [20]. The dysregulation of lncRNAs has been implicated in various diseases, such as cancer, cardiovascular diseases, and neurological disorders [21]. Recent research has underscored the vital importance of lncRNAs in the development and progression of VCI [22,23]. Research has revealed that lncRNAs affect the progression of VCI by influencing the formation and function of blood vessels [23], regulating inflammatory responses [24], and participating in the damage and repair of neuronal cells, highlighting their potential as potential therapeutic targets for VCI [25]. Studying lncRNAs is helpful for understanding the molecular mechanisms of VCI and for innovating novel therapeutic approaches. This article aims to comprehensively discuss the involvement of lncRNAs in the pathologies and potential therapy of VCI. Additionally, we suggest that targeting immune responses with tailored vaccinations might offer fresh perspectives in diagnosing and treating VCI.

## 2. Pathophysiological Mechanisms of VCI

VCI is a type of cognitive decline caused by blood vessel dysfunction in the brain. Its pathophysiological mechanism is complex and involves multiple factors and processes, including vascular lesions [26], changes in cerebral blood flow [27], nerve damage, and metabolic disorders. The dysregulation of cerebral perfusion is a prevalent cause underlying VCI. Perfusion is the fundamental process in which blood circulates through an intricate network of tiny vessels within tissue to facilitate the crucial exchange of oxygen and other essential molecules [28]. Maintaining this process is crucial in the brain for sustaining homeostasis and ensuring the proper functioning of the neurovascular unit (NVU) [29]. Proper perfusion is essential for the NVU’s operation, ensuring the brain’s health and proper function. Perturbations in perfusion, as seen in VCI, can lead to compromised blood flow to the brain, resulting in tissue damage and cognitive decline. In the following sections, we summarize the pathological changes of VCI and potential targeted interventions to mitigate its effects.

### 2.1. Dysfunction in Endothelial Cells and the Breakdown of the Blood–Brain Barrier

Cerebral endothelial cells (ECs), in conjunction with tight junction complexes and the end feet of astrocytes, are essential in establishing and preserving the integrity of the blood–brain barrier (BBB) [30]. These elements collaborate to precisely control the movement of cells and molecules between the blood and the brain’s parenchyma via specialized transporters [31]. Additionally, cerebral ECs are dynamic in regulating various vascular functions. They control blood coagulation within vessels and maintain vascular tone. Additionally, they facilitate neurovascular coupling by releasing contracting agents like endothelin-1 [32] and thromboxane, as well as relaxing factors such as nitric oxide [33], prostacyclin, and the endothelium-derived hyperpolarizing factor [34].

Cerebral ECs are pivotal in managing cerebral blood flow (CBF). They control CBF primarily by producing nitric oxide through the enzymatic conversion of L-arginine catalyzed by endothelial nitric oxide synthase (eNOS) [35]. Consequently, any abnormalities in nitric oxide production or eNOS activity are critical indicators of endothelial dysfunction. Research has shown that factors like aging and hypertension can modify eNOS, shifting its function from solely producing nitric oxide to producing both nitric oxide and superoxide anion. This dual production results in the production of peroxynitrite, a potent and cytotoxic oxidant [36]. Simultaneously, research indicates that the bioavailability of nitric oxide decreases during the aging process [37]. In vascular diseases such as atherosclerosis and arteriosclerosis, the production of endothelial nitric oxide is reduced, further diminishing its bioavailability [38]. Nitric oxide plays a crucial role in dilating vascular smooth muscle cells. It participates in the regulation of vascular tension to maintain cerebral blood flow [39]. As a result, a decrease in nitric oxide levels and its bioavailability results in the compromised regulation of cerebral blood flow. Alternatively, a deficiency in nitric oxide also diminishes the S-nitrosylation process of neuronal calpain, consequently leading to an increase in calpain activity. This heightened activity of calpain triggers the activation of cyclin-dependent kinases, which in turn results in the phosphorylation of tau proteins [40]. The accumulation of excessively phosphorylated tau is a key characteristic of AD, which is also associated with VCI [41].

In VCI, the BBB is highly likely to be compromised. The primary role of the BBB is to shield neurons from detrimental elements in the systemic circulation and preserve the internal environment of the brain. This disruption allows the entry of damaging substances like proteins, toxins, and inflammatory agents into the brain tissue. These substances can directly inflict damage upon neuronal cells or indirectly cause harm by triggering inflammatory responses. These deleterious effects further exacerbate the neuropathological conditions associated with VCI. The weakened integrity of the BBB plays a pivotal role in the sequence of events that result in neuronal dysfunction and cognitive deterioration. When cerebral blood flow decreases, sustained hypoperfusion causes endothelial dysfunction and pericyte degeneration, ultimately damaging the BBB [42]. Following the compromise of the BBB, there is an increased expression of matrix metalloproteinases (MMP)-2, MMP-3, and MMP-9, leading to the disintegration of the basement membrane and a decrease in tight junction proteins such as claudin-1, claudin-3, claudin-5, and claudin-12 [43]. This process further intensifies the breakdown of the BBB. The breakdown of the BBB acts as an early indicator of cognitive impairment [44]. In patients with early-stage small vessel disease (SVD), this breakdown typically manifests as a reduced expression of tight junction proteins between cells [45]. A similar decrease in proteins such as claudin-5, occludins, and zonula occludens-1 is also observed in the cortical areas of those suffering from Alzheimer’s disease [46]. Interestingly, elevated levels of tight junction proteins in the peripheral blood have been detected in individuals with coronary heart disease, stroke, and intracranial hemorrhage, with particularly higher concentrations of occluding and claudin-5 in these conditions [28]. These increased levels could signify endothelial dysfunction in patients with VCI, suggesting a breach in the integrity of the BBB. The compromise of the BBB leads to oxidative stress, the invasion of cells, and the infiltration of inflammatory agents, along with the penetration of neurotoxic substances from the blood [47]. These blood-derived neurotoxic substances include thrombin, low-density lipoprotein (LDL) cholesterol, albumin, fibrinogen, and plasminogen [43]. Albumin infiltration causes vasogenic edema, disrupting the brain’s microcirculation and subsequently affecting cerebral blood flow. Cytokines produced by inflammatory responses also promote the disruption of the BBB.

Moreover, the escalated production of superoxide anion by various enzymes diminishes the bioavailability of nitric oxide [48]. Identifying this reduction is of critical importance, as it can disrupt the regulation of cerebral blood flow (CBF) and compromise the brain’s oxygen supply. This, in turn, may lead to cognitive decline. Additionally, the decreased availability of nitric oxide influences the permeability of the BBB by altering tight junction proteins, subsequently heightening the risk of cerebral microbleeds [49].

### 2.2. Neuronal and Neuroglial Degeneration

Ischemic conditions, inflammatory responses, and oxidative stress collectively lead to the deterioration and eventual demise of neurons and glial cells. Damages to neurons are directly caused by inflammatory cytokines released from activated microglia and astrocytes [50]. MMP-2 and MMP-3 damage oligodendrocytes, resulting in a reduction of trophic support and subsequent degeneration of neurons [51]. Additionally, the ability of endothelial trophic factors to support oligodendrocyte precursor cells is suppressed by oxidative stress. This disruption leads to impaired oligodendrocyte production and contributes to myelin deterioration and damage to white matter [52]. During this process, myelin basic proteins and neurofilament light chains (NfL) are released [53]. Simultaneously, fibrosis occurs in the vessel walls due to the remodeling of the extracellular matrix. Cellular edema, together with fibrosis, contribute to alterations in white matter. These changes are observable as white matter hyperintensities (WMHs) on MRI scans. These changes result in a disconnection syndrome, characterized by executive impairment and psychomotor retardation [54].

### 2.3. Dysregulation of Neurovascular Unit Homeostasis

Maintaining healthy blood vessels and optimal CBF control is essential for normal brain functioning due to the brain’s constant energy demands and limited reserves. The NVU is an intricate structure, incorporating various vascular elements including pericytes, cerebral endothelial cells, and vascular smooth muscle cells, as well as an array of glial cells—microglia, oligodendrocytes, and astrocytes—and neurons. These components collectively play a vital role in preserving the structural and functional integrity of the brain [29]. The NVU is crucial in controlling CBF, the permeability of the BBB, vascular remodeling, and neuroimmune reactions [29]. Disruptions in the NVU can lead to the impaired regulation of cerebral blood flow, breakdown of the BBB, and infiltration of inflammatory cells and neurotoxic materials into brain tissue. These alterations impair brain functions and play a role in the development of neurodegenerative diseases [43]. Growing evidence indicates that dysfunction within the NVU is a key factor in brain pathologies, encompassing both VCI and various neurodegenerative diseases [55]. Additionally, cerebral autoregulation and neurovascular coupling play crucial roles in sustaining brain function and balance. These processes highlight the significance of effectively regulating cerebral blood flow.

Cerebral autoregulation is key in maintaining a stable cerebral blood flow, enabling the brain to adjust to changes in arterial blood pressure encountered in daily life. Yet, in elderly individuals with cerebrovascular conditions, this process of autoregulation is frequently impaired [56]. This disruption can lead to diminished blood flow and vascular injuries, such as WMHs. These changes increase the likelihood of cognitive decline, dementia, and stroke, ultimately leading to a higher risk of mortality [57].

Neurovascular coupling is a critical process that adjusts CBF to meet neurons’ metabolic needs during intense neuronal activity. This process relies on synchronized interactions within active neurovascular units. These interactions lead to the rapid dilation of cerebral blood vessels and enhanced cerebral blood flow in the active areas of the brain [58]. This precise regulation of CBF guarantees a sufficient delivery of oxygen and nutrients necessary for brain functions and synaptic processes. It also helps with the removal of detrimental metabolic waste products. Yet, those suffering from SVD and AD frequently show compromised neurovascular coupling, characterized by a reduced hemodynamic reaction to neuronal stimulation [59]. Such dysfunction results in either acute or chronic decreases in cerebral blood flow, impacting the delivery of oxygen and nutrients to the brain as well as the removal of metabolic waste. Although some studies suggest that impaired neurovascular coupling in aging may be linked to cognitive dysfunction, the exact mechanism remains unknown [60]. Further research is required to comprehensively understand the extent to which neurovascular coupling influences localized brain dysfunction. Additionally, research is needed to explore its impact on the broader spectrum of cognitive disorders associated with VCI.

### 2.4. Activation of Oxidative Stress

It has been reported that different patients with MCI show differences at the cellular level in macromolecular oxidative damage, reduced antioxidant defense capabilities, and mitochondrial dysfunction upon autopsy. This indicates the involvement of redox imbalance in the disease’s progression [61]. The disruption of the BBB allows for the leakage of fibrinogen, leading to the activation of microglia and astrocytes through integrins (CD11b and CD18) and Toll-like receptors. This stimulation initiates the transcription dependent on nuclear factor κB (NF-κB), resulting in the generation of reactive oxygen species (ROS) [62]. Subsequently, oxidative stress hampers the signaling of endothelial nitric oxide, redirecting the production of nitric oxide towards the creation of superoxide. This shift not only increases ROS levels, further exacerbating oxidative stress, but also reduces the anti-inflammatory capabilities of nitric oxide [63]. Furthermore, the leakage of red blood cells caused by the compromised BBB leads to the release of neurotoxic Fe^2+^, which further contributes to the elevation of ROS. Increased ROS subsequently accelerates oxidative damage and inflammation and promotes neuroinflammation via enhancing the expression of pro-inflammatory cytokines and transcription factors [61].

### 2.5. Inflammatory Response Induction

Inflammation is considered a significant factor in the progression of VCI [64]. Reports indicate an increase in interleukin-1β (IL-1β) levels in patients suffering from VCI [65]. Analyses of blood and cerebrospinal fluid samples of patients with VaD showed elevated levels of three inflammatory markers: interleukin-6 (IL-6), C-reactive protein, and tumor necrosis factor-α (TNF-α) [66]. Damage to the NVU and the disruption of the BBB can initiate inflammatory responses [67]. The accumulation of inflammatory cells and molecules not only further damages blood vessels and neuronal cells but also potentially exacerbates the decline in cognitive function. Immune cells and inflammatory agents potentially engage with elements of the NVU, furthering the spread of damage [68]. Studies suggest that the increased permeability of the BBB results in the stimulation of angiotensin receptors on perivascular macrophages. This stimulation subsequently triggers the release of ROS, potentially leading to tissue damage [69]. The leakage of fibrinogen prompts activated microglia to generate hypoxia-inducible factor 1α, leading to the production of furin. Furin, an enzyme, initiates the activation of matrix metalloproteinases (MMP-2, MMP-3, and MMP-9), which are key players in the degradation of the BBB [53]. Additionally, these processes activate the receptors for advanced glycation end-products (RAGEs). The engagement of RAGEs with advanced glycation end-products leads to the production of ROS through the activation of nicotinamide adenine dinucleotide phosphate-oxidase. This triggers NF-κB, which then stimulates genes for various cytokines such as tumor necrosis factor (TNF), interleukin (IL)-1, IL-6, and IL-8. This cascade contributes to inflammation and the additional breakdown of the BBB [70].

The activation of microglia leads to their division into two main phenotypes: pro-inflammatory and anti-inflammatory. Pro-inflammatory microglia are stimulated by Interferon-γ, whereas anti-inflammatory microglia are induced by IL-4 and IL-13 [71]. Pro-inflammatory microglia propel the neuroinflammatory process by emitting substances like nitric oxide, TNF, IL-6, and IL-1β, contributing to neuronal harm. TNF and IL-1β also activate astrocytes, leading to further inflammatory responses. In contrast, anti-inflammatory microglia release agents such as IL-10, IL-33, TGF-β, insulin-like growth factor-1 (IGF-1), nerve-derived growth factor (NGF), and brain-derived neurotrophic factor (BDNF). These cytokines are known for their neuroprotective effects [72].

Activated astroglia differentiate into two distinct polarized phenotypes, A1 and A2 [71]. A1 astroglia are marked by their production of ROS, IL-1β, TNF, and nitric oxide, all of which play roles in neuroinflammation and neuronal damage [73]. In contrast, A2 astroglia partake in a neuroprotective sequence, releasing BDNF, which counteracts RAGE, and vascular endothelial growth factor (VEGF). VEGF not only promotes angiogenesis but also improves glucose transport to the brain and stimulates antioxidants, thereby contributing to neuroprotection [74].

### 2.6. Coagulation Dysfunction

The role of coagulation factors in the emergence and progression of cerebrovascular diseases like SVD is not well understood. Recent studies show that the activation of platelets and monocytes is commonly seen in SVD patients. This is evidenced by the elevated levels of CD40L and CD62P on platelets, an increase in platelet–monocyte complexes, and a rise in tissue factor expression in monocytes, all of which contribute to the coagulation process [75]. Willebrand factor, typically stored in cerebral endothelial cells, is released following injury to assist in coagulation and platelet adhesion [76]. When endothelial cells suffer damage due to age or vascular diseases, subendothelial matrix proteins are exposed to blood, leading to platelet adhesion and collagen accumulation. These processes are driven by endothelial von Willebrand factor (vWF) [77]. Moreover, the diminished cerebral autoregulation of blood flow and heightened perivascular edema lead to the increased expression of adhesion receptors like P-selectin, E-selectin, intercellular adhesion molecule-1 (ICAM-1), and vascular cell adhesion molecule-1 (VCAM-1), facilitating leukocyte migration and platelet adhesion [58]. This sets off a coagulation cascade involving various factors, resulting in the thrombin-mediated conversion of fibrinogen to fibrin, forming thrombi and microvascular blockages that contribute to lacune development. Thrombin also has direct neurotoxic effects, causing neuronal death, astrocytic proliferation, and microglial activation through the protease-activated receptor-1 [78]. In response to thrombosis, stable fibrin chains are degraded into D-dimer by plasmin, originating from circulating plasminogen. Plasmin further enzymatically degrades neuronal matrix proteins like laminin. Disruption in the connection between neurons and their extracellular matrix render hippocampal neurons more susceptible to cell death, thereby worsening neurological damage [79]. Therefore, coagulation factors can serve as potential biomarkers for endothelial dysfunction, offering insights into the early diagnosis and prevention of cerebral SVD and VCI [47].

The underlying mechanisms of VCI’s pathophysiology unveil an intricate interplay among the vascular, nervous, and immune systems. Understanding these intricate interactions is crucial for developing effective treatment and prevention strategies. The NVU plays a central role in this process, making it a critical focus for future research and therapeutic intervention. This comprehensive understanding could lead to more targeted and effective approaches to mitigate the progression of VCI.

## 3. Basic Science of lncRNA

### 3.1. The Classification of lncRNA

LncRNAs are broadly classified into five categories based on their proximity to protein-coding genes: intergenic, intronic, antisense, semantically overlapping, and bidirectional lncRNAs [80]. Intergenic lncRNAs (lincRNAs), originating from DNA segments between genes, can impact neighboring genes by modulating their promoters or enhancers [81]. Intronic lncRNAs, derived from within gene introns, are known to influence the transcription and splicing of coding genes through various transcriptional mechanisms [82,83]. Antisense lncRNAs are produced from antisense strands of protein-coding genes, while sense lncRNAs come from the coding strands, including exons, and may overlap or cover protein-coding genes [84,85]. Bidirectional lncRNAs, characterized by transcription in opposite directions at each end, primarily engage in DNA transcription with functions akin to those of protein-coding entities [86]. The function of lncRNAs is usually influenced by their location, sequence, and secondary structure.

### 3.2. The Mechanism of lncRNA

LncRNAs are generally classified into four groups based on their action mechanisms, impacting transcriptional regulation, post-transcriptional regulation, chromatin modifications, and other functions. In transcriptional regulation, lncRNAs can influence promoters via transcriptional interference and chromatin remodeling. In addition to transcriptional interference and chromatin remodeling, lncRNAs play a crucial role in chromatin modifications, such as DNA methylation and histone modifications (acetylation, methylation). These interactions can alter the chromatin state, making it more or less accessible for transcription, thereby affecting gene expression. LncRNAs interact with chromatin-modifying enzymes, such as histone acetyltransferases (HATs) and histone deacetylases (HDACs), to regulate the post-translational modification of histones. These interactions facilitate the dynamic regulation of chromatin architecture, influencing gene accessibility and expression profiles [17,87,88]. Regarding post-transcriptional regulation, lncRNAs primarily control splicing and translation: those affecting mRNA splicing may operate by binding to or regulating splicing factors, or by directly hybridizing with mRNA sequences to obstruct splicing [89,90]. LncRNAs involved in translation regulation can attach to translation factors [91] or ribosomes. Additionally, lncRNAs have been found to engage in direct siRNA mechanisms [92,93] or interact with miRNAs [94,95,96,97]. Beyond transcriptional and post-transcriptional regulation, lncRNAs can also influence protein localization [97], telomere replication [98], RNA interference, and other mechanisms.

## 4. The Role of lncRNAs in VCI

**Figure 1:**
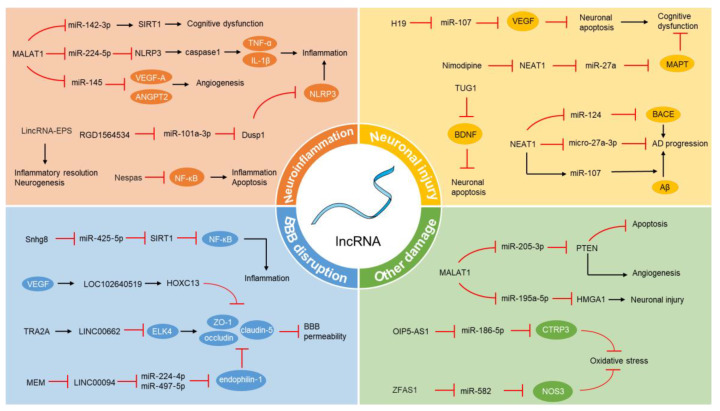
The regulation of lncRNA in the pathophysiological mechanisms of VCI. LncRNA, long non-coding RNA; VCI, vascular cognitive impairment; SIRT1, sirtuin 1; TNF-α, tumor necrosis factor-α; IL-1β, interleukin-1β; VEGF, vascular endothelial growth factor; ANGPT2, angiopoietin-2; NF-κB, nuclear factor κB; VEGF, vascular endothelial growth factor; BDNF, brain-derived neurotrophic factor; AD, Alzheimer’s disease; BACE1, beta-site amyloid precursor protein cleaving enzyme 1; Aβ, amyloid β peptide; KLF4, Kruppel-like transcription factor 4; ZO-1, zonula occludens-1; BBB, blood–brain barrier; MEM, memantine; PTEN, phosphatase and tensin homolog; HMGA1, high mobility group AT-hook 1; CTRP3, C1q/TNF-related protein 3; NOS3, nitric oxide synthase 3; The black arrows represent stimulation or upregulation, while the red prohibition symbols represent inhibition or downregulation.

While research has shown that lncRNAs are important in the development and advancement of VCI, studies focusing on lncRNAs in relation to VCI are still relatively scarce. However, the pathophysiological processes involved in the progression of VCI are also present in other diseases, where the role of lncRNAs has been more extensively studied. For example, recent research has demonstrated the regulatory role of lncRNAs in coronary artery disease (CAD) within human coronary artery smooth muscle cells (SMCs). LncRNAs in SMCs have been found to be enriched near CAD-related transcription factors (TFs) and genetic loci, suggesting their potential significance in the disease [99]. Insights gained from the function of lncRNAs in these other conditions can provide valuable references for understanding and potentially targeting lncRNAs in VCI. This cross-disease analysis might uncover common pathways and mechanisms where lncRNAs exert their influence, offering new avenues for research and therapeutic strategies in VCI. The regulatory role of lncRNA in VCI is illustrated in Figure 1.

### 4.1. Role of lncRNA in Neuronal Injury and Repair

The H19 gene is capable of producing 2.3 KB of non-coding RNA, classified as an imprinted lncRNA [100]. This gene is active during embryonic development and becomes less expressed postnatally. The potential role of H19 in the development and progression of vascular cognitive disorders is not yet fully understood. To explore this, a series of in vivo and in vitro studies have been conducted to examine H19’s involvement in neuronal apoptosis and hypoxic–ischemic cognitive impairment. Research findings suggest that H19 interacts with and diminishes mir-107 expression, which in turn enhances VEGF expression, helping to prevent neuronal apoptosis and mitigate cognitive deficits. In models of ischemic hypoxic brain injury in rats, the overexpression of H19 was found to reduce neuronal death and improve cognitive function. The results of cellular experiments indicate that the overexpression of H19 in neurons increased B-cell lymphoma-2 expression and decreased B-cell lymphoma-2-associated X, as well as total and cleaved caspase-3 expressions [101]. Thus, H19 presents a promising molecular target for translational research in VCI treatment, paving the way for future drug therapies.

TUG1, a 7.1 KB lncRNA, was initially identified through genome scans and noted for its increased expression in developing mouse retinal cells following taurine treatment [102]. It is among the first lncRNAs associated with human diseases and plays a crucial role in regulating cellular processes like proliferation, differentiation, and apoptosis [103,104,105]. While most current research on TUG1 focuses on its role in human cancers, where it is known to encourage cancer cell growth, movement, and invasiveness, some studies also link TUG1 to neurodegenerative disease pathologies [106]. Earlier research has demonstrated that aerobic exercise can mitigate VCI by elevating BDNF levels. Specifically, aerobic exercise downregulated NF-κB, consequently decreasing the expression of miR-503. BDNF is a direct target of miR-503, and following the downregulation of miR-503 expression, BDNF levels were upregulated [107]. In this context, a study found that silencing TUG1 in HT22 cells reduced hippocampal neuronal apoptosis by enhancing BDNF expression under oxygen–glucose deprivation. The experiments demonstrate that TUG1 can bind to the BDNF protein. Therefore, aerobic exercise could potentially alleviate cognitive deficits and decrease hippocampal apoptosis in VCI models by downregulating TUG1 expression [102]. In conclusion, the relationship between TUG1 and VCI requires further investigation, yet it holds potential as a prospective therapeutic target.

The lncRNA NEAT1 consists of two variants: NEAT1v1 (3.7 KB) and NEAT1v2 (23 KB), both of which are implicated in a range of diseases including viral infections, cancer, Parkinson’s disease, and others [108]. Alzheimer’s disease, often a late-stage development in VCI, sees NEAT1 playing a critical role in its onset and progression. Ke et al. [109] indicated that lncRNA NEAT1, by binding miR-107, intensifies Aβ-induced neuronal damage, thus accelerating Alzheimer’s disease development. Specifically, in cells treated with Aβ, NEAT1 expression is enhanced. Knocking down NEAT1 attenuates the Aβ-induced inhibition of cell viability, as well as the increase in cell apoptosis and p-Tau levels. NEAT1 acts as a sponge for miR-107, reducing the abundance of miR-107, thereby exacerbating neuronal damage. Meanwhile, Zhao et al. [110] proposed that NEAT1’s interaction with the miR-124/BACE1 axis could make it a novel target for Alzheimer’s disease treatment. In AD mouse models, NEAT1 is significantly upregulated, leading to a significant downregulation of miR-124. BACE1, as a downstream target of miR-124, is upregulated, thereby contributing to the progression of AD. NEAT1 is also known to promote Alzheimer’s by suppressing micro-27a-3p. In AD rat and cell models, NEAT1 expression is upregulated, while micro-27a-3p expression is downregulated. Furthermore, there is an increase in the apoptosis rate and a decrease in cell viability. Levels of amyloid-beta protein, BACE1 protein, APP protein, Tau protein and its phosphorylation, and caspase-3 protein and its cleaved form are upregulated [111]. In the context of subarachnoid hemorrhage (SAH), a type of cerebral bleed leading to severe brain damage and often resulting in cognitive impairment, studies have found that nimodipine can ameliorate cognitive impairment post-SAH in rats via the lncRNA NEAT1/Mir-27a/MAPT axis. Specifically, it has been found that nimodipine leads to the downregulation of NEAT1, consequently resulting in the upregulation of miR-27a and downregulation of MAPT. Under these conditions, the apoptosis of brain tissue cells is inhibited, brain cell activity is enhanced, ultimately repairing ischemic neuronal damage [112]. Therefore, in subsequent treatment, NEAT1 can promote the functional recovery of cognitive impairment caused by some diseases. The role of lncRNA in neuronal injury and repair is summarized in Table 1.

### 4.2. Role of lncRNA in Neuroinflammation

Metastasis-associated lung adenocarcinoma transcript 1 (MALAT1), synthesized by RNA polymerase II, is regulated at both the transcriptional and post-transcriptional stages [113]. While its link to cancer has been a primary focus in recent years, emerging studies also connect MALAT1 to chronic conditions like stroke [114] and type 2 diabetes mellitus (T2DM) [115]. Research has shown that lncRNAs, including MALAT1, can regulate specific microRNAs such as miR-224-5p, miR-216b-5p, and miR-485-5p, affecting their expression levels. This regulatory effect was highlighted in studies by Ping Du et al. [116], where blocking the MALAT1/miR-224-5p/NLRP3 pathway was found to reduce hippocampal inflammation in T2DM, particularly regarding the activation of microglial NLRP3 inflammasomes in neuroinflammation and neurodegeneration. Specifically, MALAT1 promotes NLRP3 expression by acting as a competitive endogenous RNA and sequestering miR-224-5p. The overexpression of miR-224-5p significantly inhibits the expression levels of NLRP3, caspase 1, TNF-α, and IL-1β. MiR-224-5p reduces microglial inflammation activation by regulating NLRP3 expression, ultimately impacting the NLRP3/IL-1β pathway in the hippocampus. Additionally, MALAT1 has been found to alleviate cerebral ischemia–reperfusion injuries and cognitive dysfunction by modulating the mir-142-3p/SIRT1 axis [117], and to promote microvascular endothelial cell angiogenesis by targeting Mir-145 [118]. In both in vivo and in vitro models of ischemia–reperfusion injury, MALAT1 is downregulated in a time-dependent manner, accompanied by the increased expression of miR-142-3p and decreased expression of Sirtuin 1 (SIRT1). MALAT1 negatively regulates the expression of miR-142-3p, and SIRT1 is a target gene of miR-142-3p. The overexpression of MALAT1 reduces the levels of TNF-α, IL-6, IL-1β, ROS, and MDA in PC12 cells damaged by OGD/R injury and increases the activity of superoxide dismutase (SOD) and catalase (CAT). Additionally, MALAT1 overexpression inhibits OGD/R-induced cell necrosis and apoptosis while promoting cell proliferation. Thus, MALAT1 presents itself as a potential therapeutic target in clinical settings, though its interactions with miRNA warrant further exploration.

Studies have shown that in rat models of middle cerebral artery occlusion (MCAO) and cells subjected to oxygen–glucose deprivation/reperfusion (OGD/R), there is a decrease in the expression of the lncRNA RGD1564534. However, its elevated expression has been found to mitigate neuronal harm, cognitive impairment, and damage to white matter and small vessels in MCAO rats. RGD1564534 functions by competitively binding with miR-101a-3p, thereby diminishing the interaction between miR-101a-3p and Dusp1 and increasing Dusp1 expression in neurons. This activity promotes mitochondrial autophagy, reduces NLRP3 inflammasome activity, and prevents neuronal apoptosis induced by OGD/R. Thus, as a lncRNA, RGD1564534 appears to play a vital protective role in the onset and advancement of VCI, influencing mitochondrial autophagy and inflammatory responses. This reveals its potential as a novel molecular target for VCI treatment strategies [119].

LncRNA mediates neuroinflammation after ischemic stroke [16]. In ischemic stroke patients and models, the expression pattern of lncRNA XIST initially decreases and later increases. There is a negative correlation between serum levels of lncRNA XIST in ischemic stroke patients and the extent of their neurological impairment. XIST regulates the levels of the pro-angiogenic factor integrin α5 (itgα5) and the anti-inflammatory Kruppel-like transcription factor 4 (KLF4) by interacting with microRNA-92a (miR-92a), suggesting its role in modulating angiogenesis and anti-inflammatory activities in ischemic strokes. Silencing lncRNA XIST impairs angiogenesis post-stroke and intensifies cerebral vascular damage, leading to larger infarctions and more severe neurological deficits, indicating its protective function in vascular integrity and brain tissue recovery [120]. Overactive microglia, which release excessive inflammatory factors, significantly contribute to ischemic stroke-induced brain damage. Research shows that the high levels of Gm4419 promote the phosphorylation of IκBα by physically binding to it, leading to increased nuclear levels of NF-κB, thereby activating the transcription of TNF-α, IL-1β, and IL-6. Conversely, the knockdown of Gm4419 in OGD/R astrocytes acts as an inhibitor of NF-κB, indicating a protective role of Gm4419 downregulation against OGD/R injury [121]. Meanwhile, the expression of lncRNA-1810034E14Rik is reduced in ischemic stroke, but its overexpression lowers inflammatory cytokine levels, inhibits microglial activation, and reduces the phosphorylation of p65 [122]. Furthermore, the lncRNA Nespas, which increases in ischemic brain tissue, appears to play an anti-inflammatory and anti-apoptotic role in microglia post OGD stimulation and in ischemic stroke models by inhibiting the TRIM8-associated K63-linked polyubiquitination of TAK1 [123]. Extracellular vesicles have been identified as potential carriers for drug delivery across the blood–brain barrier, opening new avenues for treating central nervous system diseases [124]. Zhang et al. constructed a biomimetic vesicle targeting microglia called LincRNA-EPS. Studies have shown that these microglia-targeted biomimetic vesicles can promote the resolution of inflammation and neurogenesis after a stroke, offering a hopeful therapeutic strategy for utilizing lncRNA in the diagnosis and management of neurological disorders [125]. The role of lncRNA in neuroinflammation is summarized in Table 2.

### 4.3. Role of lncRNA in Endothelial Function and the Blood–Brain Barrier

Researchers employed single-nucleus RNA sequencing to examine the transcriptomic profile of hippocampal endothelial cells in T2DM mice. They found significant changes in the transcriptome of these cells, including enriched RNA coding and non-coding pathways related to endothelial signaling, neuroinflammation, endothelial barrier disruption, and neurodegenerative changes. These molecular alterations were linked to the dysfunction of the BBB through neuroimaging techniques, suggesting that lncRNAs might be involved in T2DM-associated neuroendothelial and barrier damage, which could relate to vascular cognitive impairment (VCI). Additionally, the transcriptomic characteristics of diabetes showed strong parallels with the gene expression patterns observed in AD and VCI patients. These insights support the idea that neuroinflammation and damage to endothelial cells and barriers, driven by T2DM, are critical in the cognitive deterioration linked to this condition. LncRNAs, functioning as regulatory molecules, could be crucial in this mechanism, presenting potential new targets for the treatment of VCI [126].

The Western diet (WD) and hyperlipidemia may influence vascular diseases, dementia, and cognitive disorders by altering gene expression. Studies have found that in mice fed with a WD, there is a notable variation in the expression of numerous genes, with about 10% linked to non-protein coding RNAs, mainly lncRNAs. This finding indicates the possible involvement of lncRNAs in vascular abnormalities and cognitive impairment triggered by the WD, thereby contributing to the progression of VCI. The alteration in lncRNA expression due to a high-fat diet might influence the molecular mechanisms governing brain microvessels, thereby affecting cognitive abilities. This provides a molecular explanation for the increased dementia risk associated with hyperlipidemia [127].

In the AD context, the long non-coding RNA LINC00662 plays a role in regulating the permeability of the BBB. When microvascular endothelial cells are exposed to β-amyloid, LINC00662 expression increases. Conversely, reducing LINC00662 in the AD environment leads to decreased BBB permeability. LINC00662 influences the BBB by suppressing the expression of ETS domain protein 4 (ELK4), which binds to the promoters of tight junction proteins ZO-1, occludin, and claudin-5 to enhance their expression. Therefore, inhibiting LINC00662 in AD conditions can enhance ELK4 levels, increase tight junction protein concentrations, and thus improve BBB integrity. This indicates the potential of targeting LINC00662 and its regulatory pathways for VCI treatment [128]. Additionally, memantine (MEM), an N-methyl-D-aspartate (NMDA) receptor antagonist, is widely used in AD therapy. Studies have found that under certain conditions, treatment with MEM reduces the expression of LINC00094, suggesting that MEM may exert its therapeutic effects partly by regulating LINC00094. Further research revealed that LINC00094 inhibits Endophilin-1 expression by interacting with miR-224-4p/miR-497-5p. This action leads to increased levels of ZO-1, occludin, and claudin-5, effectively decreasing the permeability of the blood–brain barrier in the AD microenvironment. Hence, LINC00094 could be a viable target for VCI treatment strategies [129].

Recent research has also confirmed the influential role of lncRNAs in controlling blood–brain barrier permeability and neural recovery in cases of ischemic stroke [22]. Studies have found that in ischemic stroke models, VEGF increases the expression of LOC102640519 and HOXC13, which significantly contributes to the disruption of the BBB. LOC102640519 acts to enhance HOXC13 expression, which subsequently leads to a reduction in the expression of ZO-1, occludin, and claudin-5 in the OGD/R model. These tight junction proteins are key factors in maintaining BBB integrity [130]. In chronic cerebral ischemia models, the lncRNA Snhg8 was found to promote neuronal apoptosis. However, in microglia and brain microvascular endothelial cells (BMECs) treated with MCAO and OGD, Snhg8 expression decreases. Enhancing Snhg8 expression not only reduces microglial inflammatory responses induced by ischemia but also lessens BMEC damage both in vitro and in vivo. This indicates Snhg8’s protective role in mitigating ischemic brain injury and maintaining blood–brain barrier integrity. Through bioinformatics analysis, it was revealed that Snhg8 functions as a competitive endogenous RNA, sponging miR-425-5p and promoting microglial inflammation and BMEC injury via the sirtuin1 (SIRT1)-mediated NF-κB signaling pathway [131]. Additionally, the role of lncRNAs is also significant in hemorrhagic stroke contexts. Blnc1 is upregulated after intracerebral hemorrhage (ICH), and it regulates its function by affecting BBB cell vitality, migration, apoptosis, permeability, and inflammatory response. Inhibiting Blnc1 shows promising therapeutic benefits in reducing neural damage and inflammation caused by ICH via the activation of the PPAR-γ/SIRT6/FoxO3 signaling pathway. This finding may provide important clues for developing new strategies to treat ICH [132]. Under ICH conditions, the lncRNA Snhg3 is found to be elevated and impacts the function of the blood–brain barrier. It activates the TWEAK/Fn14/STAT3 signaling pathway, leading to dysfunction in cerebral microvascular cells in an ICH rat model. Specifically, the overexpression of Snhg3 increases the expression of the TWEAK protein and its receptor Fn14, thereby activating downstream neuroinflammatory pathways such as STAT3 and enhancing the secretion of MMP-2/9. Down-regulated Snhg3 levels can enhance recovery from neural damage and inflammation due to ICH, indicating its potential as a therapeutic target [133]. The involvement of lncRNA in endothelial function and blood–brain barrier integrity is detailed in Table 3.

### 4.4. Role of lncRNA in Pathogenic Protein Deposition

β-secretase 1 (BACE1) is a key enzyme in amyloid accumulation, which is a critical pathogenic feature in various neurodegenerative disorders, especially AD. A lncRNA named BACE1AS regulates the expression of BACE1. BACE1AS is transcribed in the opposite direction of the BACE1 gene and acts to increase BACE1 expression. The dysregulation of BACE1AS is associated with upregulated BACE1 activity in several human disorders, including Alzheimer’s disease, Parkinson’s disease, heart failure, and MCI, indicating a potential influential role of BACE1AS in the progression of these diseases [23]. In patients with MCI, the expression level and activity of BACE1 are elevated, higher than those in healthy controls and AD patients, implying that BACE1, and possibly BACE1AS, might be used to differentiate between MCI and AD, offering potential for understanding and diagnosing early pathological changes related to vascular cognitive impairment [134].

### 4.5. Role of lncRNA in Neurovascular Unit

The role of lncRNA in the NVU is less studied in VCI. However, the relationship between lncRNA and NVU in ischemic stroke can provide a reference for researchers [135]. Research indicates that lncRNA MALAT1 has diverse roles in different components of the NVU. In endothelial cells, lncRNA MALAT1 reduces cell apoptosis and promotes angiogenesis, thereby playing a protective role in the endothelium. Specifically, knocking down MALAT1 promotes OGD-induced cell apoptosis, reduces cell viability, and inhibits caspase-3 activation. MALAT1 acts as a competitive endogenous RNA (ceRNA) for miR-205-3p and further regulates PTEN expression, thereby protecting against OGD-induced cell apoptosis [136]. However, in neurons and astrocytes, MALAT1 promotes cell death through cell apoptosis and inflammation. In this condition, knocking down MALAT1 can reverse OGD/R-induced cell apoptosis and ER stress. MALAT1 upregulates High Mobility Group AT-hook 1 (HMGA1) by sponging miR-195a-5p, promoting neuronal damage induced by OGD/R [137].

### 4.6. Role of lncRNA in Oxidative Stress

Studies have found that in cases of ischemic stroke, the increased expression of lncRNA OIP5-AS1 acts protectively against neuronal damage. It achieves this by mitigating microglial/macrophage inflammation and oxidative stress caused by MCAO/R through the mechanism of miR-186-5p sponging and the subsequent activation of CTRP3 [138]. Similarly, lncRNA ZFAS1 guards against neuronal harm in cerebral ischemia–reperfusion (I/R) injury, influencing inflammation, oxidative stress, cell apoptosis, and nitric oxide levels by regulating miR-582-3p. Specifically, ZFAS1 acts as a molecular sponge for miR-582-3p, negatively regulating the expression of miR-582-3p, thereby inhibiting inflammation, oxidative stress, and cell apoptosis in OGD/R PC12 cells, while increasing NO levels. It can be used to treat damage caused by ischemic hypoxia [139]. However, some lncRNAs are upregulated after ischemic stroke, exacerbating oxidative stress and ischemic hypoxic injury. For instance, in ischemic stroke, lncRNA KCNQ1OT1 expression levels are increased. It exacerbates ischemia–reperfusion injury by targeting and suppressing miR-140-3P. Specifically, the overexpression of miR-140-3p significantly alleviates inflammation, oxidative stress, and cell apoptosis in OGD/R. KCNQ1OT1 directly binds to and inhibits the expression of miR-140-3p. All the cytoprotective effects of miR-140-3p overexpression are hindered by the co-overexpression of KCNQ1OT1. Additionally, there is a direct interaction between miR-140-3p and hypoxia-inducible factor 1α (HIF-1α), which is suppressed by the upregulation of KCNQ1OT1 in OGD/R [140]. Similarly, SNHG14 is found to be elevated in a mouse model of MCAO. Reducing the levels of SNHG14 lessens the severity of ischemic brain damage by inhibiting inflammation and oxidative stress via the miR-199b/AQP4 pathway. Specifically, the knockdown of miR-199b reversed the effect of SNHG14 knockdown on ischemic injury in OGD-stimulated BV2 cells. The overexpression of AQP4 abolished the effect of miR-199b on ischemic injury in OGD-treated BV2 cells. SNHG14 indirectly regulates AQP4 expression by sponging miR-199b [141]. The role of lncRNA in pathogenic protein deposition, the neurovascular unit, and oxidative stress is summarized in Table 4.

## 5. Exploring the Intersection of lncRNA and Immunization in VCI

mRNA vaccines represent a groundbreaking approach to vaccination, distinct from traditional protein-based vaccines [142,143]. Traditional vaccines typically involve administering inactivated or attenuated pathogens or their proteins, whereas mRNA vaccines leverage the host cell’s own machinery to induce immune responses. This innovative vaccine category delivers synthetic mRNA molecules encoding specific antigens directly into host cells. Once inside the cell, the mRNA instructs the cellular machinery to produce the target antigen, mimicking the natural infection process without causing disease. The immune system then recognizes these antigens as foreign substances and mounts a robust immune response, including antibody production and the activation of T cells, to counter potential future infections. In mRNA vaccines, lncRNAs play a crucial regulatory role.

Unlike traditional protein-focused vaccines, lncRNA vaccines tap into the regulatory functions of these non-coding transcripts [144]. Their importance lies in modulating gene expression and signaling pathways, offering a precise way to shape immune responses. By binding to transcriptional regulatory factors or transcription factors, lncRNAs can enhance or inhibit the transcription of target genes, thereby regulating antigen production. This regulatory mechanism allows for precise control of the antigen expression level in vaccines according to the need, achieving the accurate modulation of immune responses. Through interaction with key signaling molecules or the regulation of the expression of signaling pathway modulators, lncRNAs can adjust the responsiveness and functional state of immune cells. This fine-tuning can optimize the types and intensity of immune responses, thereby enhancing the immunogenicity and efficacy of vaccines. This approach signifies a shift towards tailored and targeted immunization, marking a significant advancement in vaccine strategies. The status of lncRNA vaccine development reveals a burgeoning field with promising prospects. Researchers are actively exploring key lncRNA targets within the vaccine strategy. This dynamic landscape signifies a shift toward precision medicine, aiming to harness the regulatory potential of lncRNAs for enhanced immune modulation. Ongoing investigations into these specific lncRNA targets hold the key to advancing the next generation of vaccines.

The application of lncRNA vaccines presents a promising avenue for addressing specific pathological aspects of VCI. By leveraging the regulatory role of lncRNAs, these vaccines hold potential in modulating neuroinflammation [145], oxidative stress [146], and other processes related to VCI. LncRNAs have been found to hold potential value as vaccines in other diseases. Many MHC class I-associated peptides originate from small open reading frames within lncRNA genes. When presented to the immune system, lncRNA-derived peptides drive effective antigen-specific CD8 T lymphocyte responses, resulting in a significant delay in tumor growth. Therefore, lncRNA-encoded peptides represent immunogenic epitopes that can be utilized as cancer vaccine candidates [147]. The lncRNA DIO3OS is significantly downregulated in hepatocellular carcinoma. Studies have shown that DIO3OS interacts with the NONO protein, limiting NONO-mediated ZEB1 mRNA nuclear export, thereby inhibiting the progression of hepatocellular carcinoma. This research provides valuable candidate drugs for the targeted therapy of hepatocellular carcinoma [148]. Endocrine therapy is the frontline treatment for estrogen receptor (ER)-positive breast cancer patients. However, resistance to endocrine therapy drugs remains a challenge. Estrogen-induced LINC02568 promotes the development and resistance of ER-positive breast cancer through both trans and cis mechanisms. Targeting LINC02568 with antisense oligonucleotides (ASOs) significantly inhibits the in vitro growth of ER-positive breast cancer cells and in vivo tumor formation. This suggests that targeting LINC02568 could serve as a potential therapeutic avenue clinically [149]. Long non-coding RNAs (lncRNAs) also play a crucial role in infectious diseases. Snhg1 has been found to interact with the conserved vesicle transport protein Vps13D, regulating IL-7 signaling and promoting the generation of memory CD8 T cells. This discovery provides strong support for enhancing vaccine efficacy during the COVID-19 pandemic and controlling sustained outbreaks [150]. Another study has shown that lncRNA NR_003508 regulates the RIPK1 inhibition of Mycobacterium tuberculosis-induced necroptosis by sponging miR-346-3p. This finding suggests that lncRNAs could serve as promising therapeutic targets for tuberculosis [151]. The development of lncRNA vaccines offers new possibilities for modulating these pathological processes. By designing vaccines targeting specific lncRNAs, we can intervene in neuroinflammation and oxidative stress processes, thereby alleviating or preventing the development of VCI. These vaccines can regulate the expression levels of lncRNAs in immune cells and neuronal cells, affecting the activity of related signaling pathways and inflammation responses. This precise regulatory mechanism provides new avenues and strategies for treating VCI, offering the potential for more effective treatment options and better prospects for recovery for VCI patients.

Developing lncRNA vaccines for neurological disorders poses unique challenges that warrant careful consideration. Overcoming issues related to delivery, specificity, and the intricacies of neural environments requires innovative solutions. However, these challenges also present opportunities for advancing research and refining vaccine development strategies. Firstly, the treatment of neurological disorders requires overcoming the BBB restriction to ensure the effective delivery of drugs or vaccines to neural tissues. Innovative delivery methods, such as utilizing extracellular vesicles, nanoparticles, liposomes, or polymer nanoparticles, can help overcome BBB limitations and achieve the effective delivery of lncRNA vaccines into the nervous system [124]. Moreover, specific delivery vehicles can be designed to enable the targeted release of lncRNA vaccines within neural tissues, thereby enhancing therapeutic efficacy. Secondly, the unique properties of neural tissues should be taken into consideration when treating neurological disorders. For example, the complex connections of neurons and interactions between neurons and glial cells should be carefully considered. Therefore, targeted strategies are crucial to ensure the precise delivery of lncRNAs to targeted neural cells or regions. By designing specific targeting sequences or utilizing neuro-specific enhancers, the precise targeting of specific cell types or regions within the nervous system can be achieved, thereby enhancing vaccine efficacy and reducing side effects [125]. Additionally, the treatment of neurological disorders also requires overcoming the complexity and unpredictability of the neural environment. Innovative vaccine design and optimization strategies, such as combining multiple therapeutic targets or modulating the expression levels of lncRNAs in different cell types, can enhance the therapeutic efficacy of vaccines for neurological disorders. Furthermore, in-depth understanding and ongoing clinical trials of neurological disorders are also crucial for driving the application of lncRNA vaccines in neurological diseases. Navigating these obstacles with strategic insights can unlock the full potential of lncRNA vaccines in the realm of neurological diseases.

## 6. Conclusions

In the realm of VCI, lncRNA, a specific type of non-coding RNA, holds significant importance. These multifunctional molecules are key players in a range of central nervous system disorders, particularly within the complex neuroimmune and inflammatory cascades. This article comprehensively summarized the pathophysiological aspects of VCI, including endothelial cell and blood–brain barrier dysfunction, neuronal and glial cell degeneration, neurovascular unit dysregulation, inflammation, oxidative stress, and coagulation disorders. These elements form a comprehensive framework for grasping the complexities of VCI and identifying possible intervention strategies. As research in this field is still in its infancy, we expanded the discussion to include insights from lncRNA studies in other neurological diseases, offering a broader perspective and potential parallels with VCI. Excitingly, there are current studies utilizing lncRNAs in clinical therapy. A clinical trial from the Chinese PLA General Hospital is prospectively predicting the immunotherapy response of gastric cancer based on circulating exosomal LncRNA-GC1 biopsy (ClinicalTrials.gov Identifier: NCT05334849). Another clinical study from Southeast University investigates the effect and mechanism of lncRNA NBR2 regulating endothelial pyroptosis by targeting GSDMD in sepsis (ClinicalTrials.gov Identifier: NCT04427371). Additionally, lncRNAs are also being utilized as diagnostic biomarkers for various diseases, such as oral squamous cell carcinoma (ClinicalTrials.gov Identifier: NCT05708209), liver cancer (ClinicalTrials.gov Identifier: NCT05840133), thyroid cancer (ClinicalTrials.gov Identifier: NCT03469544), bladder cancer (ClinicalTrials.gov Identifier: NCT05270174), and lung cancer (ClinicalTrials.gov Identifier: NCT03830619).

We believe it is essential to consider how insights into lncRNA function and VCI intersect with broader themes in medical research. Specifically, we should examine their implications for immunization strategies, sequencing technologies, and vaccine development. Exploring the function of lncRNAs in regulating immune reactions and inflammatory processes presents an encouraging avenue for developing specific immunization approaches. By understanding the specific lncRNAs involved in the pathological processes of VCI, researchers can design vaccines or develop immunotherapies aimed at modulating these critical molecular players. Furthermore, the advancement of NGS technologies has been a boon for lncRNA research, allowing for the comprehensive identification and quantification of lncRNAs in various conditions. The future of VCI research and potential therapeutic strategies are likely to be heavily influenced by the continued evolution and application of NGS, which enable a more detailed and nuanced understanding of lncRNA profiles and interactions. In summary, the convergence of lncRNA research with immunization strategies and advanced sequencing technologies like NGS represents a promising avenue for addressing the complex challenges of VCI. As we continue to explore the complex roles of lncRNAs in VCI and utilize advancements in immunization and sequencing technologies, we can expect to gain new insights and develop innovative approaches. These advancements will contribute to our fight against not only VCI but also other neurological disorders. The path ahead is complex but filled with potential for significant breakthroughs that could transform the landscape of VCI research and treatment.

## Figures and Tables

**Table 1:** Role of lncRNA in neuronal injury and repair.

Diseases	lncRNAs	Study Materials	Effects	Mechanisms	Reference
Ischemic stroke	H19	Rats, neuronal cells	Inhibiting neuronal apoptosis and alleviating cognitive dysfunction	Inhibiting miR-107 and upregulating VEGF	[101]
VCI	TUG1	Mice, HT22 cells	The knockdown of TUG1 reducing hippocampal neuronal apoptosis and participating in aerobic exercise-alleviated VCI	Increasing BDNF level	[102]
AD	NEAT1	SH-SY5Y, SK-N-SH cells	Aggravating Aβ-induced neuronal damage	Sponging miR-107	[109]
AD	NEAT1	Mice	Knockdown of NEAT1 showing the protective effects on cellular AD model induced by Aβ	Regulating miR-124/BACE1 axis	[110]
AD	NEAT1	SH-SY5Y cells, rat	Promoting AD	Downregulating micro-27a-3p	[111]
SAH	NEAT1	Rat	Nimodipine improving cognitive impairment after SAH in rats	Regulating the lncRNA NEAT1/miR-27a/MAPT axis	[112]

VEGF, vascular endothelial growth factor; VCI, vascular cognitive impairment; BDNF, brain-derived neurotrophic factor; AD, Alzheimer’s disease; BACE1, beta-site amyloid precursor protein cleaving enzyme 1; SAH, subarachnoid hemorrhage.

**Table 2:** Role of lncRNA in neuroinflammation.

Diseases	lncRNAs	Study Materials	Effects	Mechanisms	Reference
T2DM with OSA	MALAT1	Brain tissues	Blocking the LncRNA MALAT1 inhibiting the hippocampal inflammatory response	Blocking the lncRNA MALAT1/miR-224-5p/NLRP3 axis	[116]
Stroke	MALAT1	BMECs	Promoting angiogenesis and proliferation of BMECs under OGD conditions	Enhancing the expressions of VEGF-A and ANGPT2 by targeting miR-145	[118]
CI/R	MALAT1	Mice	Improving cerebral infarction, neurological impairment, and cognitive dysfunction in CI/R mice	Regulating the miR-142-3p/SIRT1 axis	[117]
Ischemic stroke	RGD1564534	Rat	Alleviating neuronal damage, cognitive dysfunction, as well as white matter and small vessel injuries	Binding with miR-101a-3p, increasing the expression of Dusp1 in neurons	[119]
Ischemic stroke	XIST	Mice, brain endothelial cells	Maintaining vascular integrity and promoting brain tissue repair	Regulating the expression of itgα5 and KLF4 by targeting miR-92a	[120]
Ischemic stroke	Gm4419	Microglia	Contributing to OGD/R injury of cerebral microglial cells	Phosphorylating IκB and activating NF-κB	[121]
Ischemic stroke	1810034E14Rik	Mice, microglia	Decreasing the infarct volume and alleviated brain damage	Suppressing the activation of microglial cells and inhibiting the phosphorylation of p65	[122]
Ischemic stroke	Nespas	Mice, microglia	Playing an anti-inflammatory and anti-apoptotic role	Inhibiting the TRIM8-associated K63-linked polyubiquitination of TAK1	[123]
Ischemic stroke	LincRNA-EPS	Mice, microglia	Promoting inflammatory resolution and neurogenesis	NA	[125]

MALAT1, metastasis-associated lung adenocarcinoma transcript 1; T2DM, type 2 diabetes mellitus; OSA, obstructive sleep apnea; BMECs, brain microvascular endothelial cells; OGD, oxygen–glucose deprivation; VEGF, vascular endothelial growth factor; ANGPT2, angiopoietin-2; CI/R, cerebral ischemia–reperfusion; SIRT1, sirtuin 1; itgα5, integrin α5; KLF4, Kruppel-like transcription factor 4; miR-92a, microRNA-92a; OGD/R, oxygen glucose deprivation/reoxygenation; NF-κB, nuclear factor κB; NA, no accessible data in the study.

**Table 3:** Role of lncRNA in endothelial function and the blood–brain barrier.

Diseases	lncRNAs	Study Materials	Effects	Mechanisms	Reference
AD	LINC00662	BMECs	Knockdown of TRA2A or LINC00662 decreasing BBB permeability	TRA2A increasing the stability of LINC00662, and LINC00662 decreasing ELK4 expression through SMD pathway to downregulate the expression of ZO-1, occludin, and claudin-5	[128]
AD	LINC00094	hCMEC/D3	MEM used widely for AD therapy, and silencing LINC00094 enhancing the effect of MEM on decreasing BBB permeability in AD microenvironment	Reduction of LINC00094 inhibiting endophilin–1 expression by upregulating miR–224–4p/miR–497–5p and promotes the expression of ZO–1, occludin, and claudin–5 in AD conditions	[129]
Ischemic stroke	LOC102640519	Mice, mBMECs	Administration of VEGF upregulating LOC102640519 and aggravating BBB permeability	Positively regulating the expression of HOXC13, thus negatively regulating the expression of ZO–1, occludin, and claudin–5	[130]
Ischemic stroke	Snhg8	Mice, BMECs	Increasing ZO–1 and occludin, promoting angiogenesis, inhibiting microglial activation after MCAO, and reducing inflammatory factor IL–1β, IL–6, and TNF–α	Serving as a ceRNA by sponging miR–425–5p to regulate sirtuin1-mediated NF-κB pathway	[131]
ICH	Blnc1	Mice, BMVECs	Suppressing cell viability and migration but increasing permeability, apoptosis, and inflammation in vitro, and its suppression ameliorating nerve injury, brain edema, BBB permeability, and the levels of inflammatory cytokines in vivo	Exacerbating nerve injury and inflammatory response through PPAR-γ/SIRT6/FoxO3 pathway	[132]
ICH	Snhg3	Mice, BMVECs	Snhg3 inhibition improving cell proliferation and migration and reducing cell apoptosis and monolayer permeability in vitro, and improving behavioral scores, BBB integrity, brain water content and cell apoptosis in vivo	Regulating cerebral microvascular cell injury through Snhg3/TWEAK/ Fn14/STAT3/MMP-2/-9 pathways	[133]

AD, Alzheimer’s disease; BMECs, brain microvascular endothelial cells; TRA2A, transformer 2 alpha; BBB, blood–brain barrier; ELK4, ETS domain protein 4; SMD pathway, Staufen-mediated mRNA decay pathway; ZO-1, zonula occludens-1; hCMEC, human cerebral microvascular endothelial cells; MEM, memantine; mBMECs, mouse brain microvascular endothelial cells; VEGF, vascular endothelial growth factor; HOXC13, homeobox C13; MCAO, middle cerebral artery occlusion; IL, interleukin; TNF–α, tumor necrosis factor-alpha; ceRNA, competing endogenous RNA; NF-κB, nuclear factor-kappa B; ICH, intracerebral hemorrhage; BMVECs, brain microvascular endothelial cells; MMP, matrix metalloproteinase.

**Table 4:** Role of lncRNA in pathogenic protein deposition, neurovascular unit, and oxidative stress.

Diseases	lncRNAs	Study Materials	Effects	Mechanisms	Reference
MCI	BACE1AS	CSF of patients with MCI	Positively regulating the expression of BACE1	NA	[134]
Ischemic stroke	MALAT1	Endothelial cells	Reducing cell apoptosis and promoting angiogenesis	Sponging miR-205-3p and modulating PTEN expression	[136]
Ischemic stroke	MALAT1	Neurons, astrocytes	Promoting OGD/R-induced neuronal injury	Sponging miR-195a-5p to upregulate HMGA1	[137]
Ischemic stroke	OIP5-AS1	Rats, microglia	Protecting against neuronal injury from MCAO/R-induced microglial/macrophage inflammation and oxidative stress	Sponging miR-186-5p and activating CTRP3	[138]
Ischemic stroke	ZFAS1	Rats, PC12	Improving neuronal injury and inhibiting inflammation, oxidative stress, and apoptosis	Sponging miR-582 and upregulating NOS3 expression	[139]
Ischemic stroke	KCNQ1OT1	PC12	Exacerbating cerebral ischemia–reperfusion injury	Binding to miR-140-3p, thus interfering its direct interaction with HIF-1α	[140]
Ischemic stroke	SNHG14	Mice, microglia	Knocking down SNHG14 alleviating ischemic brain injury, inhibiting inflammation and oxidative stress	Regulating miR-199b/AQP4 axis	[141]

MCI, mild cognitive impairment; BACE1, β-secretase 1; CSF, cerebrospinal fluid; NA, no accessible data in the study; OGD/R, oxygen–glucose deprivation/reperfusion; HMGA1, high mobility group AT-hook1; MCAO/R, middle cerebral artery occlusion/reperfusion; HIF-1α, hypoxia-inducible factor-1α.

## Data Availability

Not applicable.

## Abbreviations

AD	Alzheimer’s disease
Aβ	amyloid β peptide
BACE1	β-secretase 1
BBB	blood–brain barrier
BDNF	brain-derived neurotrophic factor
BMECs	brain microvascular endothelial cells
CBF	cerebral blood flow
ceRNAs	competitive endogenous RNAs
ECs	endothelial cells
ELK4	ETS domain protein 4
eNOS	endothelial nitric oxide synthase
I/R	Ischemia–reperfusion
ICAM-1	intercellular adhesion molecule-1
ICH	intracerebral hemorrhage
IGF-1	insulin-like growth factor-1
IL	interleukin
IL-1β	interleukin-1β
IL-6	interleukin-6
itgα5	integrin α5
KLF4	Kruppel-like transcription factor 4
LDL	low-density lipoprotein
lincRNAs	intergenic lncRNAs
lncRNAs	long non-coding RNAs
MALAT1	metastasis-associated lung adenocarcinoma transcript 1
MCAO	middle cerebral artery occlusion
MCI	mild cognitive impairment
MEM	memantine
miR-92a	microRNA-92a
miRNAs	microRNAs
MMP	matrix metalloproteinases
ncRNA	non-coding RNA
NfL	neurofilament light chain
NF-κB	nuclear factor κB
NGF	nerve-derived growth factor
NGS	next-generation sequencing
NMDA	N-methyl-D-aspartate
NVU	neurovascular unit
OGD	oxygen–glucose deprivation
OGD/R	oxygen–glucose deprivation/reperfusion
RAGE	receptors for advanced glycation end-products
ROS	reactive oxygen species
SAH	subarachnoid hemorrhage
SIRT1	sirtuin1
SVD	small vessel disease
T2DM	type 2 diabetes mellitus
TNF	tumor necrosis factor
TNF-α	tumor necrosis factor-α
VaD	vascular dementia
VCAM-1	vascular cell adhesion molecule-1
VCI	vascular cognitive impairment
VEGF	vascular endothelial growth factor
vWF	von Willebrand factor
WD	Western diet
WMHs	white matter hyperintensities
